# Nerve-End Cells in the Dental Pulp

**Published:** 1920-08

**Authors:** J. Howard Mummery


### NERVE-END CELLS IN THE DENTAL PULP

BY J. HOWARD MUMMERY, M.R.C.S.

In a previous paper of Dr. Mummery's it was shown that the nerves of the tooth pulp do not terminate at the inner margin of the dentine, but actually enter the dentinal tubules with the dentinal fibrils or processes of the odontoblast, and are distributed within the hard tissue of the dentine.

By the methods of preparation then used the neurofibril was demonstrated as a continuous uninterrupted fiber, its continuity with the axis cylinder of the medullated nerves of the pulp being evident.

By certain modifications of these methods preparations were obtained in which the neurofibrils were stained throughout their whole length as continuous beaded fibers both in the pulp and in the dentine. In these preparations the nerve-end cells remain transparent and invisible; the neurofibrils appear to pass direct to the dentine, their continuity with the medúllated fibres of the pulp being clearly seen.

The point of first importance is that the neurofibrils in the plexus beneath the odontoblasts do not pass direct to the dentine, but enter small nerve-end cells or corpuscles which lie in a row along the bases of the odontoblast cells.

The peripheral distribution of the nerves in the dentine is thus seen to take place from definite nerve-end bodies situated at the lower margin of the odontoblast layer, each end cell contributing a process to the dentine which would appear to represent the axon of the cell, and numerous other processes apparently corresponding to the dendrons of a nerve cell; these dendrons form a network around the odontoblasts. The individual end cells have mostly a stellate form and are collected into groups slightly separated from one another. The distal process of the end cell is usually unbranched and passes up between the odontoblasts in a wavy course, to enter the dentine with the dentinal fibril. They are often seen to communicate by parallel cross-branches, giving a kind of basket-like appearance to the cluster.

In many parts of the periphery of the pulp the cells can not be seen, probably owing to the thickness of the section, or to the plane of the section not being quite parallel to them, and unless the reduction of the gold has been, very complete they do not appear at all, but apparently remain quite transparent, although the fibers with which they are connected are darkly stained and their connection with the dentine plainly shown.

Many preparations were examined with a high power (1/16th and No. 4 ocular) to ascertain if any of the nerve fibers in the deep plexus were distributed to the odontoblast layer without the intervention of the end cells; no evidence was obtained of this in sections where the end cells were distinctly stained. Sometimes one of the larger end cells was seen with fine processes radiating from it on all sides, and there was no apparent process to the dentine —but these were scarce, and in most places the beaded delicate fibers forming a network around the odontoblasts were distinctly seen to be given off from lateral processes of the end cells. The absence of the distal process in some of the radiating cells may possibly be due to the section not having passed in the plane of this process, for in most places it appears as if each end cell is provided with both sets of processes.

Sometimes the processes to the dentine appear to be wound together in spirals, and there is often an appearance of fibrillation in the cell body.

There is great variation in the size of these end cells, but many which appear to be single are probably groups of cells. The size of the nerve fiber or bundle of neurofibrils passing to the dentine is also very variable.

The bundles are especially large at the crown of the pulp, where there is a much larger area of dentine to be supplied. The bundles probably divide and subdivide within the dentinal tubes and their branches. Small enlargements or nodes are seen upon the fibers distributed around the odontoblasts, but are not present on the distal or dentine side. These strongly beaded fibers, first described as the marginal plexus—derived from the dentritic process of the nerve cells—are evidently a portion of the network of fibers surrounding the odontoblasts and extending to the dentine margin.

Great difficulty was experienced in procuring a definite reduction of the gold in the neurofibrils within the tubes clearly in the dentine. The fibers were seen to pass some little way into the tubules as definite dark-stained fibrils accompanying the dentinal fibril, but soon ceased to be uninterrupted, and were continued as lines of black dots.

In some fortunate preparations, however, the reduction had reached the right stage within the tubes, and even close to the cement beneath the granular layer of tubes the fibrils are seen as winding black fibers within the tubes. In several places the nerve fiber and the dentinal fibril can be seen in the same tube—the nerve fiber appearing as a delicate black line accompanying the brown-stained dentinal fibril. In the very fine terminal branches of the dentinal tubes at the cement margin only a delicate uniform dotting can be seen in these gold preparations, and this appearance is also visible in the canaliculi of the cement with which these fine branches are continuous. But while these appearances would seem to indicate that there is a direct nerve communication between the pulp and the cement, we can not definitely prove this until the fiber in this situation is seen to be clearly continuous, since in all metallic impregnations deceptive appearances may arise from deposits.

It is, however, noticeable that in these finer divisions of the tube the dotting is uniform, and does not show the variation in size of the particles usually exhibited by the irregular deposit which so often occurs in the larger tubes.

After several attempts the nerve terminations were successfully shown at the enamel margin of the dentine. To make evident the relations of the nerve fibers to the enamel, it was necessary to stain ground sections of teeth which had not been decalcified. Such sections were ground fairly thin, then subjected to the prolonged action of a weak gold chloride solution, reduced in the same manner as the decalcified preparations, then ground as thin as possible on a stone with water. Here and there they pass for a short distance into the enamel, apparently in the interprismatic spaces, where they terminate in small bulbous enlargements.

Where "spindles" are present in the enamel, especially at the apices of the cusps, fine winding fibers are sometimes seen within them, but these spindles do not appear to contain definite nerve organs. From the preparations made, however, one would be inclined to consider the penetration of these bodies by nerve fibers more as due to the fact that nerve fibers in the tubes would be likely to penetrate into any open space with which the tubes communicate than as constituting definite nerve-end organs.

Minute beads or enlargements are seen upon the neurofibrils in the tubes in many places, and in some, under high magnification, delicate threads are seen to be continued from these enlargements into the finer divisions of the dentinal tubes.

Are all these sensory fibers, or are some of them trophic and supplied to the secreting cells of the odontoblast layer?

The mode of termination in the pulp described above from peripheral nerve-end organs or cells, is not a usual one for sensory nerves, and does not appear to be met with elsewhere in the body.

Healthy dentine is sensitive. A fractured tooth where the dentine is exposed and the pulp not opened is intensely sensitive, and the operation of cutting down the surface of a ''live'' tooth as soon as the enamel has been removed and the dentine reached the grinding of the latter is felt acutely. In the excavation of a carious tooth, little pain is felt during the excavation of the diseased portion, but when the last layer of carious substance is raised from the healthy dentine beneath, the pain is usually very acute.

These observations lead to the conclusion that the processes prolonged to the dentine from the nerve cells in the pulp are principally sensory fibers.

We know also that the pulp of the tooth when accidentally exposed is acutely sensitive. We should there imagine that both sensory and trophic fibers would be distributed to the pulp and also to the dentine which contains within the tube a protoplasmic prolongation of the odontoblast cell, the maintenance of the functions of which would probably necessitate a nervous supply.

### METHODS OF PREPARATION

Fixation.—Teeth immediately after extraction are placed in formol (the commercial 40 per cent. solution of formaldehyde) and water, or formol and normal salt solution, preferably 4 per cent. of formol. This is after a few days changed to a 10 per cent. solution, and the teeth kept in this for at least a fortnight. Teeth with unfinished roots may be placed in the fixing solution without being cut; those with completed roots should be sawn in two at the neck.

Decalcification.—The decalcifying solution is that of Guido Fischer, and consists of 53.3 per cent. solution of formic acid in distilled water—Fischer adds 5 per cent. of formol to the solution. This is not a very rapid decalcifying agent; teeth are generally completely softened in a fortnight.

Washing.—The teeth must be washed in running water for twenty-four hours. They are then cut into pieces of the required size and form and transferred to distilled water for a few minutes.

Impregnation.—The pieces taken from the distilled water are suspended by threads in a large quantity of a weak solution of gold chloride (1 in 5,000). Each piece should be suspended in at least 100 c.c. of the solution, in which it is left in the dark for from four to seven days, according to its size. On removal from the gold solution it is washed for a few moments only in distilled water before employing the reducing agent.

Reduction.—The pieces are placed in a 20 per cent. solution of caustic soda for four minutes. On removal from this solution they are rinsed in water and placed in a 10 per cent. solution of potassium carbonate for from half an hour to an hour. This is then drained off and the pieces are placed in a 10 per cent. solution of potassium iodide for five to ten minutes. As soon as they are seen to darken they are removed to water, placed in gum for twelve hours and cut on the freezing microtome.

The sections are then dehydrated and mounted in carnsal (propylic) balsam. This mounting medium gives very clear definition and clearing agents are dispensed with as they can be mounted direct from absolute alcohol. —*Dental Record.*

